# Cognitive function and its determinants in elderly Indonesians residing in long-term care: Insights from a cross-sectional study

**DOI:** 10.12688/f1000research.158490.2

**Published:** 2025-08-22

**Authors:** Etty Rekawati, Winda Eriska, Utami Rachmawati, Dwi Nurviyandari Kusuma Wati, Junaiti Sahar, Arief Andriyanto, Jing-Jy Wang, Sri Susanty, Faizul Hasan

**Affiliations:** 1 Department of Community Nursing, Faculty of Nursing, Universitas Indonesia, Depok, West Java, Indonesia; 2 Department of Community Nursing, Universitas Bina Sehat PPNI Mojokerto, Mojokerto, Indonesia; 3 Department of Nursing, College of Medicine, National Cheng Kung University, Tainan City, Tainan City, Taiwan; 4 Alzheimer’s Disease Research Center, National Cheng Kung University Hospital, Tainan, Taiwan; 5 Nurse Professional Education Study Program, Faculty of Medicine, Universitas Halu Oleo, Kendari, South East Sulawesi, Indonesia; 6 Faculty of Nursing, Chulalongkorn University, Bangkok, Bangkok, Thailand

**Keywords:** Cognitive function; elderly individuals; risk factors

## Abstract

**Background:**

Multiple medical conditions arising from reduced physical and physiological functioning, including cognitive decline, manifest in older persons. This study aims to examine the relationship between cognitive function and associated risk factors in older persons living in long-term care facilities in Indonesia.

**Methods:**

This study involved 350 elderly individuals residing in long-term care institutions. A cross-sectional design utilizing an analytical survey methodology was implemented. Data were gathered via interviews employing a demographic questionnaire and the Montreal Cognitive Assessment (MoCA). Statistical analysis was conducted using SPSS (version 23).

**Results:**

Univariate analysis demonstrated significant correlations between cognitive performance and gender, ethnicity, level of education, medical history, subjective memory issues, smoking habits, alcohol consumption, dietary intake of fruits and vegetables, and employment history (p < 0.05). Higher education (OR = 0.69, 95% CI: 0.56–0.84) and reduced subjective memory complaints (OR = 0.29, 95% CI: 0.20–0.44) correlated positively with enhanced cognitive function, but alcohol intake (OR = 6.79, 95% CI: 2.42–19.1) correlated with impaired cognitive function.

**Conclusions:**

the level of education, subjective memory complaints, and alcohol intake are substantially correlated with cognitive performance in older persons residing in long-term care facilities. Evaluating demographic characteristics in elderly individuals can assist healthcare professionals in the early detection of cognitive impairment, facilitating prompt interventions in long-term care environments.

## Introduction

By 2030, one in six individuals worldwide will be senior citizens. In Indonesia, life expectancy improved from 68.6 years in 2018 to 71.8 years in 2022, with an anticipated increase of 72.2 years for the period 2030–2035. ^1^ The 2022 Indonesia National Health Survey indicated that 10.5% of the population comprises elderly persons. ^2^ The aging population has transitioned the illness burden from infectious diseases and malnutrition to chronic ailments such as diabetes, hypertension, neoplasms, and coronary heart disease. ^3^ These alterations hinder daily functioning and augment economic dependency. ^4^ Moreover, physical, mental, and emotional deterioration intensifies reliance, impairing social interactions, self-care, and health management. ^5^

Mental changes in older persons encompass transformations in personality, memory, and cognitive ability, shaped by socio-demographic, physical, and psychological factors, ^6– 9^ with loneliness, social isolation, ^7, 10, 11^ and late-life mental diseases. ^6^ With the expansion of the older adult demographic, cognitive impairment has become increasingly common. ^8^ Cognitive function denotes the capacity to uphold responsibilities and social interactions, and its deterioration impedes engagement with family and community, imposing a burden on caregivers and communities. ^12^

Numerous individual factors affect cognitive decline, including age, ^13^ gender, ^14– 16^ level of education, ^17– 19^ genetics, and medical history. Chronic disorders include hypertension, ^20, 21^ diabetes, ^22– 24^ cardiovascular diseases, ^25, 26^ gastritis, ^27– 29^ and depression ^30, 31^ exacerbate cognitive impairment. Environmental factors, including social engagement and physical activity, significantly influence outcomes. ^32– 34^

Research has revealed multiple indicators strongly linked to motor-cognitive risk, including extremity functional limits, activities of daily living (ADL) impairment, fatigue, and hypertension. ^35^ Age, medical history, depression, and resilience are determinants of cognitive function. ^36^ Timely recognition and intervention of these factors are crucial to avert or alleviate cognitive impairment in elderly individuals. ^37^ Neuropsychological evaluations, like the Mini Mental State Examination (MMSE) and the Montreal Cognitive Assessment (MoCA), are essential instruments for identifying cognitive deficits, with MoCA demonstrating heightened sensitivity. ^5^

Long-term care in Indonesia offers help for anyone requiring extended assistance, especially the elderly or individuals with disabilities. Nonetheless, there is a lack of particular data regarding the population of older adults residing in Long-Term Care Institutions (LTCI). The swift expansion of the elderly demographic has heightened the demand for long-term care insurance, influenced by evolving social dynamics and diminished familial capacity to offer care. Nevertheless, no previous research has investigated the correlation between cognitive function, medical history, and related risk factors in elderly individuals, impeding healthcare practitioners’ capacity to execute preventive measures and inform families.

Unlike previous regional studies that predominantly examined community-dwelling elderly, this study focuses on older persons residing in LTCIs, a population with distinct vulnerabilities that has rarely been explored in Indonesia. By addressing this gap, the study provides novel insights that not only contextualize cognitive health in institutional care within Indonesia but also contribute to the broader global discourse on aging, long-term care, and cognitive decline. This cross-sectional study aims to examine the relationship between cognitive function and associated risk factors among older persons in long-term care institutions in Indonesia.

## Methods

### Study setting and participants

This analytical cross-sectional study was performed in the major Long-Term Care Institutions in Jakarta, Indonesia, from February to April 2023. It comprised older people (≥60 years) devoid of eyesight or hearing impairments. Participants with visual or hearing impairments were excluded because the MoCA-Ina requires intact sensory abilities to ensure valid measurement of cognitive function. Deficits in vision or hearing could interfere with item completion and lead to misinterpretation of test scores as cognitive impairment. Participants were selected using purposive sampling. All residents in the two LTCIs who met the eligibility criteria were screened and included in the study. This approach allowed the inclusion of the entire eligible population. During the preliminary phase, health records were examined to ascertain eligible participants, contingent upon the nursing home’s consent. A total of 350 elderly people participated in the study. Specifically, two major long-term care institutions in Jakarta participated in this study. In addition, the study follow the STROBE guideline ( https://www.equator-network.org/).

### Variables and measures

*Demographic characteristics*

Data were obtained via in-person interviews. Demographic variables encompassed age, duration of residence in long-term care institutions and nursing homes, gender, religion, ethnicity, relationship status, level of education, medical history, subjective memory issues, tobacco use, alcohol intake, daily consumption of fruits and vegetables, employment background, utilization of mobility aids, and living situation.

*The montreal cognitive assessment (MoCA-Ina)*

The Indonesian adaptation of the Montreal Cognitive Assessment (MoCA-Ina) was utilized, with all procedures executed in the native language, Bahasa Indonesia, to guarantee participant comfort. MoCA-Ina has exhibited robust reliability and validity, evidenced by a Cronbach’s alpha of 0.976, signifying exceptional internal consistency. ^38^ Cognitive function, the principal variable, was evaluated using the 30-point MoCA-Ina, with scores ≥11 signifying strong cognitive function and scores <11 denoting low cognitive function. MoCA-Ina is a reliable and sensitive instrument for identifying moderate cognitive impairment in elderly individuals in Indonesia. ^39^

### Data collection procedure

Ten trained enumerators aided senior citizens in completing the questionnaire. Authorization was secured from the facility director, and the study protocols were comprehensively elucidated. Subsequent to approval, enumerators obtained consent from participants, who signed consent forms upon their agreement to participate. Eligible participants were apprised of the study’s objectives, advantages, and methodologies prior to receiving the questionnaire. Participation was optional and confidential, with each session lasting 15 to 20 minutes.

### Ethical consideration

Prior to the investigation, this study has approval from the institutional review board (IRB) Committee of Universitas Indonesia with approval number of KET-168/UN2.F12.D1.2.1/PPM.00.02/2022 on June 21, 2022. This study adhered to the Declaration of Helsinki ( https://www.wma.net/policies-post/wma-declaration-of-helsinki-ethical-principles-for-medical-research-involving-human-subjects/). Written informed consent was obtained from participants prior joining the study and were apprised of the study’s objectives, benefits, and methods.

### Data analysis

All the information collected were input into Microsoft Excel and analyzed utilizing SPSS version 23 (IBM SPSS Statistics Version 29, 2023). Descriptive data were displayed as numerical values and percentages. Chi-square tests were performed to evaluate the relationships between independent factors and cognitive function. Univariate analysis utilizing logistic regression calculated unadjusted odds ratios and their respective 95% confidence intervals (CIs) for all relevant risk factors, with significance established at p < 0.05. Variables were included in the multivariate logistic regression model if they were theoretically relevant based on prior research on cognitive decline and/or demonstrated statistical significance in univariate analysis at a threshold of p < 0.25. This approach ensured that the model captured both conceptually important and empirically supported factors.

## Results

### Demographic characteristics

The frequency distribution of all demographic and clinical variables is presented in Table 1. No missing data were identified in the dataset, as all 350 participants provided complete information across the included variables.

Overall, the mean age of participants was 68.9 years (SD 7.01), with a median of 68. The mean duration of residence in the long-term care institution (LTCI) was almost 3 years (SD 3.6), with a median of 3 years. The average cognitive function score was 12.8 (SD 7.25), with a median of 11.

Table 1 reveals that the predominant demographic of respondents consisted of early seniors (60-68 years old), with 186 participants (53.1%), whereas 164 participants (46.9%) were older. Over fifty percent resided in the institution for fewer than three years (227 participants, 64.9%). Female enrollment was greater, with 193 individuals (55.1%), in contrast to male enrollment, which comprised 157 persons (44.9%). The majority of participants identified as Muslim (303 participants, 86.6%), with 148 (42.3%) reporting good health, and hypertension as the most prevalent health concern (50 individuals, 14.3%). The majority consisted of married individuals (138 participants, 39.4%), those of Javanese ethnicity (135 participants, 38.6%), and elementary school graduates (107 participants, 30.6%).

**Table 1.** Demographic characteristics of older adults in nursing homes (n = 350).

	Frequency	(%)
**Length of stay in nursing home (years)**		
≤ 3	227	64.9
> 3	123	35.1
**Gender**		
Male	157	44.9
Female	193	55.1
**Religion**		
Islam	303	86.6
Christianity/Protestantism	35	10
Catholicism	4	1.1
Buddhism	5	1.4
Hinduism	2	0.6
Other	1	0.3
**Ethnic group**		
Javanese	135	38.6
Sundanese	51	14.6
Betawi	97	27.7
Sumatran	44	12.6
Chinese	9	2.6
Other	14	4.0
**Marital status**		
Married	138	39.4
Divorced dead	79	22.6
Divorced alive	38	10.9
Single	95	27.1
**Education level**		
No schooling	52	14.9
Did not graduate from elementary school	93	26.6
Graduated from elementary school	107	30.6
Graduated from junior high school	44	12.6
Graduated from high school	47	13.4
Diploma/Other higher education school	7	2.0
**Disease history**		
Hypertension (HT)	50	14.3
Diabetes mellitus (DM)	6	1.7
Cholesterol	2	0.6
Heart failure	2	0.6
Depression	5	1.4
Mental disorders	65	18.6
Strokes	8	2.3
Arthritis	14	4.0
Cataracts	5	1.4
Gastritis	3	0.9
**Subjective memory complaint**		
Very bad	3	0.9
Bad	77	22.0
Medium	183	52.3
Very good	87	24.9
**Smoking**		
Yes (active)	79	22.6
Yes (passive)	8	2.3
Stopped	47	13.4
Does not smoke	216	61.7
**Consumption of alcoholic beverages**		
Which, now	3	0.9
Which, once was	38	10.9
Never	309	88.3
**Consumption of fruits and vegetables every day**		
Yes	317	90.6
No	33	9.4
**Employment history**		
Formal/professional work	10	2.9
Informal work	335	95.7
Retired	5	1.4
**Use of walking aids**		
Yes	44	12.6
No	306	87.4
**Living arrangement**		
Living with family	142	40.6
Alone	208	59.4
**MoCA**		
High cognitive function	199	56.9
Low cognitive function	151	43.1

MoCA: Montreal Cognitive Assessment.

Participants indicated moderate subjective memory problems (183 participants, 52.3%), with the majority abstaining from alcohol consumption (309 participants, 88.3%) and not engaging in smoking (216 participants, 61.7%). A considerable percentage of participants ingested fruits and vegetables daily (317 participants, 90.6%). Nearly all participants were informal workers (335 individuals, 95.7%). Fifty-nine point four percent of participants had previously lived alone (208 participants), whereas forty point six percent lived with family (142 participants). Additionally, fifty-nine point four percent of participants (208 individuals) did not utilize walking assistance. A total of 199 participants (56.9%) achieved higher scores on the MoCA, whilst 151 participants (43.1%) attained lower scores.

### Univariate analysis

Table 2 illustrates the association between participant characteristics and MoCA scores, which function as an indicator of cognitive ability. The findings demonstrate that multiple factors significantly correlate with MoCA scores, including gender, ethnicity, level of education, medical history, subjective memory issues, smoking behaviors, alcohol intake, dietary practices (particularly fruit and vegetable consumption), and employment background.

**Table 2.** Association between cognitive levels and risk factors in older adults living in nursing homes.

Variable	Total	MoCA Score		*P*-Value
	(n= 350)	High cognitive function (%)	Low cognitive function (%)	
**Length of stay in nursing home**				
≤ 3 years	227 (64.9)	136 (38.9)	91 (26)	0.117
> 3 years	123 (35.1)	63 (18)	60 (17.1)	
**Gender**				
Male	157 (44.9)	104 (29.7)	53 (15.1)	**0.001**
Female	193 (55.1)	95 (27.1)	98 (28.0)	
**Religion**				
Islam	303 (86.6)	169 (48.3)	134 (38.3)	0.522
Christian	35 (10)	23 (6.6)	12 (3.4)	
Catholic	4 (1.1)	2 (0.6)	2 (0.6)	
Buddhism	5 (1.4)	3 (0.9)	2 (0.6)	
Hinduism	2 (0.6)	2 (0.6)	0 (0)	
Others	1 (0.3)	0 (0)	1 (0.3)	
**Ethnic group**				
Javanese	135 (38.6)	68 (19.4)	67 (19.1)	**0.044**
Sundanese	51 (14.6)	23 (6.6)	28 (8.0)	
Betawi	97 (27.7)	63 (18)	34 (9.7)	
Sumatra	44 (12.6)	28 (8)	16 (4.6)	
Chinese	9 (2.6)	7 (2)	2 (0.6)	
Other	14 (4)	10 (2.9)	4 (1.1)	
**Marital status**				
Married	138 (39.4)	76 (21.7)	62 (17.7)	0.851
Divorced dead	79 (22.6)	45 (12.9)	34 (9.7)	
Divorced alive	38 (10.9)	24 (6.9)	14 (4.0)	
Single	95 (27.1)	54 (15.4)	41 (11.7)	
**Education level**				
Diplomas/higher education	7 (2.0)	6 (1.7)	1 (0.3)	**0.000**
Graduated high school	47 (13.4)	33 (9.4)	14 (4.0)	
Graduated middle school	44 (12.6)	32 (9.1)	12 (3.4)	
Graduated from elementary school	107 (30.6)	65 (18.6)	42 (12.0)	
Did not graduate from elementary school	93 (26.6)	50 (14.3)	43 (12.3)	
No schooling	57 (14.9)	13 (3.7)	39 (11.1)	
**Disease history**				
Hypertension (HT)	50 (14.3)	34 (9.7)	16 (4.6)	
Diabetes mellitus (DM)	6 (1.7)	6 (1.7)	0 (0)	
Cholesterol	2 (0.6)	1 (0.3)	1 (0.3)	
Heart failure	2 (0.6)	2 (0.6)	0 (0)	
Depression	5 (1.4)	3 (0.9)	2 (0.6)	
Mental disorders	65 (18.6)	28 (8.0)	37 (10.6)	
Strokes	8(2.3)	6 (1.7)	2 (0.6)	
Arthritis	14 (4.0)	11 (3.1)	3 (0.9)	
Cataracts	5 (1.4)	4 (1.1)	1 (0.3)	
Gastritis	3 (0.9)	3 (0.9)	0 (0)	
**Subjective memory complaints**				
Very good	91 (24.5)	17 (19.1)	20 (5.3)	**0.000**
Currently	183 (52.3)	111 (31.7)	72 (20.6)	
Bad	77 (22)	20 (5.7)	57 (16.3)	
Very bad	3 (0.9)	0 (0)	3(0,9)	
**Smoking**				
Does not smoke	216 (61.7)	112 (32)	104 (29.7)	**0.03**
Quit smoking	47 (13.4)	28 (8.0)	19 (5.4)	
Passive smoker	8 (2.3)	6 (1.7)	2 (0.6)	
Active smoker	79 (22.6)	53 (15.1)	26 (7.4)	
**Alcohol consumption**				
Never	309 (88.3)	164 (46.9)	145 (41.4)	**0.000**
Yes, once	38 (10.9)	32 (9.1)	6 (1.7)	
Yes, now	3 (0.9)	3 (0.9)	0 (0)	
**Consumption of fruits and vegetables**				
Yes	317 (90.6)	187 (53.4)	130 (37.1)	**0.013**
No	33 (9.4)	12 (3.4)	21 (6.0)	
**Employment history**				
Formal/professional	10 (2.9)	8 (2.3)	2 (0.6)	**0.045**
Informal	335 (95.7)	186 (53.1)	149 (42.6)	
Retired	5 (1.4)	5 (1.4)	0 (0)	
**Use of walking aids**				
No	306 (87.4)	180 (51.4)	126 (36)	0.053
Yes	44 (12.6)	19 (5.4)	25 (7.1)	
**Living arrangement**				
Living with family	208 (59.4)	124 (35.4)	84 (24.0)	0.207
Alone	142 (40.6)	75 (21.4)	67 (19.1)	

MoCA: Montreal Cognitive Assessment.

### Multivariate logistic regression

Table 3 indicates that the model accounts for 32.1% of the variance in MoCA scores and accurately classifies 72.6% of instances. This proportion suggests that the model was able to capture nearly one-third of the determinants of cognitive function among institutionalized elderly. While this is a meaningful contribution, it also indicates that other factors not included in the model may explain the remaining variability. Upon integrating all notable predictors, three variables were recognized as significantly correlated with cognitive levels: education level (OR = 0.686; p = 0.000), subjective memory complaints (OR = 0.293; p = 0.000), and alcohol intake (OR = 6.786; p = 0.000). Older persons who abstained from drinking were 6.786 times more likely to exhibit a high cognitive level than those who consumed alcohol. Furthermore, individuals with advanced education possessed a 0.686 probability of attaining elevated cognitive capabilities as they aged. Moreover, persons devoid of subjective memory complaints exhibited a 0.293 probability of possessing elevated cognitive levels in contrast to those who indicated bad or very poor memory complaints.

**Table 3.** Multivariate logistic regression: predictive factors for cognitive levels in older adults living in nursing homes.

Predictor variables	B	SE	Wald	*p*- value	OR	95%CI Lower–Upper
Gender	-0.196	0.304	0.415	0.520	0.822	0.453–1.493
Ethnic group	-0.104	0.097	1.160	0.282	0.901	0.746–1.089
Education level	-0.377	0.106	12.716	0.000	**0.686**	**0.558–0.844**
Disease history	-0.004	0.021	0.038	0.846	0.996	0.955–1.039
Subjective memory complaints	-1.228	0.205	5.950	0.000	**0.293**	**0.196–0.437**
Smoking	-0.035	0.127	0.075	0.785	0.966	0.753–1.239
Alcohol consumption	1.915	0.527	13.203	0.000	**6.786**	**2.416–19.062**
Consumption of fruits and vegetables	0.423	0.467	0.823	0.364	1.527	0.612–3.813
Employment history	-0.126	0.736	0.029	0.864	0.864	0.208–3.732

B: beta coefficients; SE: standard error; OR: odds ratio; CI: confident interval.

## Discussion

This study revealed that older persons living in long-term care institutions (LTCIs) in Jakarta, Indonesia, displayed elevated MoCA scores, signifying enhanced cognitive performance. This study contradicts prior studies, which indicated that older persons in long-term care institutions are more prone to cognitive deterioration than those in community settings. ^40^ A nationally representative longitudinal study in China revealed that living arrangements significantly influence cognitive decline, with solitary living associated with accelerated cognitive deterioration in older men, whereas diverse living arrangements, including cohabitation with spouses and adult children, correlated with cognitive decline in older women. ^41^ Social isolation, loneliness, and restricted social involvement have been linked to reduced cognitive results in later life. ^42^ A recent meta-analysis indicated a broad spectrum of moderate cognitive impairment (MCI) prevalence among older persons in long-term care institutions, ranging from 4.0% to 87.4%, with a pooled prevalence of 21.2%. ^43^ The superior cognitive function noted in older adults in Jakarta may be ascribed to diverse social activities offered by these institutions, including entertainment gatherings, daily exercise, religious activities, and essential services, all of which improve quality of life in accordance with the Republic of Indonesia’s Social Welfare Law of 2012 concerning the care of older adults.

The research revealed educational attainment, self-reported memory issues, and alcohol intake as predictors of cognitive decline in elderly individuals residing in long-term care institutions. A meta-analysis indicated that each additional year of schooling decreases the risk of Alzheimer’s disease by 8% and dementia by 7%. ^44^ The association is exacerbated by age, as prior research suggests that schooling may alleviate racial and ethnic differences in cognitive performance among older adults. ^45^ Elevated educational attainment enhances cognitive reserve, postponing the clinical onset of Alzheimer’s disease until brain pathology is further advanced. ^46^ Thus, evaluating educational attainment in LTCIs can yield significant baseline data for the early identification of cognitive deterioration. ^47, 48^ Educational background cultivates cultural competency, augments reading proficiency, and develops problem-solving skills. ^49^

Subjective memory complaints (SMCs) are frequently observed in older persons and may signify possible cognitive impairment. ^50^ While several studies indicate that subjective memory complaints (SMCs) may not consistently align with objective cognitive impairments, ^51^ they may nonetheless be associated with anatomical alterations in the brain that facilitate cognitive deterioration. ^52^ A study in India identified correlations between tobacco use, smoking, alcohol intake, and cognitive impairment in older adults. ^53^ Alcohol intake has been demonstrated to induce brain damage by processes such as iron accumulation ^54^ and increased white matter hyperintensity volumes, ^55^ and is associated with Wernicke-Korsakoff syndrome, which negatively impacts memory and heightens the risk of cognitive impairment.

The study revealed that disease history was associated with cognitive levels, but it did not serve as a predictive variable. Cognitive performance can be affected by various factors, including age, gender, education, lifestyle choices, and the existence of chronic illnesses such as hypertension and diabetes. ^56, 57^ Studies have shown that elderly individuals with chronic conditions, especially hypertension or diabetes, may demonstrate diminished cognitive ability. ^58^ Furthermore, strokes may result in dementia, with severity influenced by factors like stroke site, volume, and pre-existing cognitive deficits. ^59^ The steady progression of cognitive decline, affected by multiple intricate factors, complicates this relationship. ^60^ Consequently, SMCs function as significant indicators of cognitive impairments and initial manifestations of Alzheimer’s disease and associated dementias.

To alleviate cognitive decline, it is essential for healthcare practitioners and family to cultivate trusting connections, encourage social engagement, and involve older persons in group activities. ^37^ Regular engagement in cognitive-stimulating activities, including adequate relaxation and sleep, is crucial, in addition to practices such as reading and media consumption. ^61^ In addition to these findings, this study provides novelty by focusing on institutionalized elderly in Indonesia, a population that has been rarely examined in prior regional research. While earlier studies have largely addressed community-dwelling older adults, our results demonstrate how the LTCI environment may shape cognitive outcomes. This contribution not only enriches the local evidence base but also offers insights relevant to global policy and practice, particularly in the design and evaluation of elderly care models in low- and middle-income countries.

This study’s limitations encompass variances in the cognitive status of older persons and its cross-sectional methodology, which constrains causal assumptions; thus, additional longitudinal investigations are essential for deeper insights. Moreover, increased sample sizes would improve analytical precision. Healthcare practitioners must prioritize initiatives that enhance cognitive function in older persons, while future research should concentrate on creating customized therapies for cognitive impairments associated with particular health problems.

## Conclusions

This research revealed educational level, subjective memory issues, and alcohol use as significant predictors of cognitive performance in older persons residing in long-term care facilities. Moreover, variables like gender, ethnicity, medical history, tobacco use, dietary intake of fruits and vegetables, and occupational background were associated with cognitive performance, underscoring the necessity for customized healthcare interventions. These findings offer significant insights into the responses of older persons to cognitive impairment risk factors, allowing nurses and healthcare professionals to formulate more effective treatment regimens. Moreover, comprehending these links helps guide personalized interventions and promote equitable health policy, ultimately enhancing care for older individuals in Indonesia.

## Ethical declaration

Prior to the investigation, this study has approval from the institutional review board (IRB) Committee of Universitas Indonesia with approval number of KET-168/UN2.F12.D1.2.1/PPM.00.02/2022 on June 21, 2022. This study adhered to the Declaration of Helsinki ( https://www.wma.net/policies-post/wma-declaration-of-helsinki-ethical-principles-for-medical-research-involving-human-subjects/). Written informed consent was obtained from participants prior joining the study and were apprised of the study’s objectives, benefits, and methods.

## Data Availability

The data behind this work can be obtained from the corresponding author (Ast. Prof. Faizul Hasan, RN, PhD, Email: faizul.h@chula.ac.th or Asc. Prof. Dr. Etty Rekawati, E-mail: rekawati@ui.ac.id) upon a reasonable request. Access to the data is restricted due to ethical issues and standards established by the Institutional Review Board (IRB) to safeguard participant confidentiality. Prospective data users must submit a written request detailing the intended purpose of data utilization and evidence of adherence to ethical norms. Approval will be contingent upon compliance with the stipulations set forth by the IRB, and applicants may be required to furnish institutional endorsement or present supplementary evidence to guarantee the proper use of the data. Zenodo Repository: STROBE checklist for ‘Cognitive Function and Its Determinants in Elderly Indonesians Residing in Long-Term Care: Insights from a Cross-Sectional Study’. https://doi.org/10.5281/zenodo.14048299 ^62^ Data are available under the terms of the Creative Commons Attribution 4.0 International license (CC-BY 4.0).

## References

[1] Indonesia Ministry of Health: *InfoDatin “Lansia Berdaya, Bangsa Sejahtera.” Report.* Jakarta: Kementerian Kesehatan RI;2024. Reference Source

[2] Indonesia Central Bureu of Statistics: *BPS. Statistik Penduduk Lanjut Usia 2022.* Jakarta:2024. Reference Source

[3] Higuchi M : Preventing non-communicable diseases in low-and middle-income countries: A literature review. *The Malaysian Journal of Nursing (MJN).* 2021;13(1):10–16. 10.31674/mjn.2021.v13i01.002

[4] Ramli R Fadhillah MN : Faktor yang mempengaruhi fungsi kognitif pada lansia. *Window of Nursing Journal.* 2020;23–32. 10.33096/won.v1i1.246

[5] Al Rasyid I Syafrita Y Sastri S : Hubungan faktor risiko dengan fungsi kognitif pada lanjut usia kecamatan Padang Panjang Timur kota Padang Panjang. *Jurnal Kesehatan Andalas.* 2017;6(1):49–54. 10.25077/jka.v6i1.643

[6] Srivastava S Sulaiman K Drishti D : Factors associated with psychiatric disorders and treatment seeking behaviour among older adults in India. *Sci. Rep.* 2021;11(1):24085. 10.1038/s41598-021-03385-7 34916551 PMC8677798

[7] Reynolds CF 3rd Jeste DV Sachdev PS : Mental health care for older adults: Rrecent advances and new directions in clinical practice and research. *World Psychiatry.* 2022;21(3):336–363. 10.1002/wps.20996 36073714 PMC9453913

[8] Barahona AJ Bursac Z Veledar E : Relationship of blood and urinary manganese levels with cognitive function in elderly individuals in the United States by race/ethnicity, NHANES 2011–2014. *Toxics.* 2022;10(4):191. 10.3390/toxics10040191 35448452 PMC9025725

[9] Susanty S Effendy DS Tosepu R : Anxiety and dementia in older adults in Indonesia. *Paper presented at: 1st International Conference Medical and Health Science Halu Oleo (IMHO 2023).* 2024. 10.2991/978-94-6463-392-4_15

[10] Rodríguez-Rey R Garrido-Hernansaiz H Collado S : Psychological impact and associated factors during the initial stage of the coronavirus (COVID-19) pandemic among the general population in Spain. *Front. Psychol.* 2020;11:1540. 10.3389/fpsyg.2020.01540 32655463 PMC7325630

[11] Susanty S Chung M-H Chiu H-Y : Prevalence of loneliness and associated factors among community-dwelling older adults in Indonesia: A cross-sectional study. *Int. J. Environ. Res. Public Health.* 2022;19(8):4911. 10.3390/ijerph19084911 35457781 PMC9025207

[12] Hutasuhut AF Anggraini M Angnesti R : Analisis fungsi kognitif pada lansia ditinjau dari jenis kelamin, riwayat pendidikan, riwayat penyakit, aktivitas fisik, aktivitas kognitif, dan keterlibatan sosial. *Jurnal Psikologi Malahayati.* 2020;2(1). 10.33024/jpm.v2i1.2428

[13] Murman DL : The impact of age on cognition. *Semin. Hear.* 2015;36:111–121. 10.1055/s-0035-1555115 27516712 PMC4906299

[14] Lin KA Choudhury KR Rathakrishnan BG : Marked gender differences in progression of mild cognitive impairment over 8 years. *Alzheimers Dement.* 2015;1(2):103–110. 10.1016/j.trci.2015.07.001 26451386 PMC4593067

[15] Wang J Xiao LD Wang K : Gender Differences in cognitive impairment among rural elderly in China. *Int. J. Environ. Res. Public Health.* 2020;17(10):3724. 10.3390/ijerph17103724 32466167 PMC7277614

[16] Levine DA Gross AL Briceño EM : Sex differences in cognitive decline among US adults. *JAMA Netw. Open.* 2021;4(2):e210169–e210169. 10.1001/jamanetworkopen.2021.0169 33630089 PMC7907956

[17] Brucki SMD Nitrini R : Cognitive impairment in individuals with low educational level and homogeneous sociocultural background. *Dement. Neuropsychol.* 2014;8:345–350. 10.1590/S1980-57642014DN84000007 29213924 PMC5619182

[18] Wang Y Wang S Zhu W : Reading activities compensate for low education-related cognitive deficits. *Alzheimers Res. Ther.* 2022;14(1):156. 10.1186/s13195-022-01098-1 36242017 PMC9563722

[19] Lövdén M Fratiglioni L Glymour MM : Education and cognitive functioning across the life Span. *Psychol. Sci. Public Interest.* 2020;21(1):6–41. 10.1177/1529100620920576 32772803 PMC7425377

[20] Ungvari Z Toth P Tarantini S : Hypertension-induced cognitive impairment: From pathophysiology to public health. *Nat. Rev. Nephrol.* 2021;17(10):639–654. 10.1038/s41581-021-00430-6 34127835 PMC8202227

[21] Canavan M O’Donnell MJ : Hypertension and cognitive impairment: A review of mechanisms and key concepts. *Front. Neurol.* 2022;13:13. 10.3389/fneur.2022.821135 35185772 PMC8855211

[22] Mallorquí-Bagué N Lozano-Madrid M Toledo E : Type 2 diabetes and cognitive impairment in an older population with overweight or obesity and metabolic syndrome: Baseline cross-sectional analysis of the PREDIMED-plus study. *Sci. Rep.* 2018;8(1):16128. 10.1038/s41598-018-33843-8 30382190 PMC6208341

[23] Munshi MN : Cognitive dysfunction in older adults with diabetes: What a clinician needs to know. *Diabetes Care.* 2017;40(4):461–467. 10.2337/dc16-1229 28325796

[24] Dove A Shang Y Xu W : The impact of diabetes on cognitive impairment and its progression to dementia. *Alzheimers Dement.* 2021;17(11):1769–1778. 10.1002/alz.12482 34636485

[25] Celutkiene J Vaitkevicius A Jakstiene S : Cognitive decline in heart failure: More attention is needed. *Card. Fail. Rev.* 2016;2(2):106–109. 10.15420/cfr.2016:19:2 28785462 PMC5490972

[26] Goh FQ Kong WKF Wong RCC : Cognitive impairment in heart failure—A review. *Biology.* 2022;11(2):179. 10.3390/biology11020179 35205045 PMC8869585

[27] Momtaz YA Hamid TA Ibrahim R : Gastritis may boost odds of dementia. *Am. J. Alzheimers Dis. Other Dement.* 2014;29(5):452–456. 10.1177/1533317513518654 24408749 PMC10852707

[28] Han M-L Chen J-H Tsai M-K : Association between Helicobacter pylori infection and cognitive impairment in the elderly. *J. Formos. Med. Assoc.* 2018;117(11):994–1002. 10.1016/j.jfma.2017.11.005 29175144

[29] Erickson LD White DS Bassett P : Cognitive function in UK adults seropositive for Helicobacter pylori. *PLoS One.* 2023;18(6):e0286731. 10.1371/journal.pone.0286731 37285350 PMC10246820

[30] Colwell MJ Tagomori H Chapman S : Pharmacological targeting of cognitive impairment in depression: Recent developments and challenges in human clinical research. *Transl. Psychiatry.* 2022;12(1):484. 10.1038/s41398-022-02249-6 36396622 PMC9671959

[31] Lam RW Kennedy SH McIntyre RS : Cognitive dysfunction in major depressive disorder: Effects on psychosocial functioning and implications for treatment. *Can. J. Psychiatry.* 2014;59(12):649–654. 10.1177/070674371405901206 25702365 PMC4304584

[32] Zhu D Al Mahmud A Liu W : Social connections and participation among people with mild cognitive impairment: Barriers and recommendations. *Front. Psych.* 2023;14:14. 10.3389/fpsyt.2023.1188887 37476544 PMC10356108

[33] Lydon EA Nguyen LT Nie Q : An integrative framework to guide social engagement interventions and technology design for persons with mild cognitive impairment. *Front. Public Health.* 2022;9:9. 10.3389/fpubh.2021.750340 35096730 PMC8795670

[34] Bae SM : The mediating effect of physical function decline on the association between social activity and cognitive function in middle and older Korean adults: Analyzing ten years of data through multivariate latent growth modeling. *Front. Psychol.* 2020;11:11. 10.3389/fpsyg.2020.02008 33391061 PMC7775571

[35] Bai A Xu W Lin Z : Prevalence and correlates of motoric cognitive risk syndrome in Chinese community-dwelling older adults. *Front. Aging.* 2022;3:3. 10.3389/fragi.2022.895138 35821814 PMC9261413

[36] Van Patten R Iverson GL Terry DP : Predictors and correlates of perceived cognitive decline in retired professional rugby league players. *Front. Neurol.* 2021;12:12. 10.3389/fneur.2021.676762 34707552 PMC8542796

[37] Mohanty M Kumar P : Multi-component interventions in older adults having subjective Cognitive Decline (SCD)—a review article. *Geriatrics.* 2023;8(1):4. 10.3390/geriatrics8010004 36648909 PMC9844291

[38] Lestari S Mistivani I Rumende M : Comparison between mini mental state examination (MMSE) and Montreal cognitive assessment Indonesian version (MoCA-Ina) as an early detection of cognitive impairments in post-stroke patients. *J. Phys. Conf. Ser.* 2017;884:012153. 10.1088/1742-6596/884/1/012153

[39] Rambe AS Fitri FI : Correlation between the Montreal Cognitive Assessment-Indonesian Version (Moca-INA) and the Mini-Mental State Examination (MMSE) in elderly. *Open Access Maced. J. Med. Sci.* 2017;5(7):915–919. 10.3889/oamjms.2017.202 29362618 PMC5771294

[40] Setiyani R Iskandar A : Cognitive impairment among older adults living in the community and in nursing home in Indonesia: A pilot study. *Dement. Neuropsychol.* 2022;16:347–353. 10.1590/1980-5764-DN-2022-0012 36619837 PMC9762390

[41] Yu Y Lv J Liu J : Association between living arrangements and cognitive decline in older adults: A nationally representative longitudinal study in China. *BMC Geriatr.* 2022;22(1):843. 10.1186/s12877-022-03473-x 36348275 PMC9644618

[42] Evans IEM Llewellyn DJ Matthews FE : Living alone and cognitive function in later life. *Arch. Gerontol. Geriatr.* 2019;81:222–233. 10.1016/j.archger.2018.12.014 30654180

[43] Chen P Cai H Bai W : Global prevalence of mild cognitive impairment among older adults living in nursing homes: A meta-analysis and systematic review of epidemiological surveys. *Transl. Psychiatry.* 2023;13(1):88. 10.1038/s41398-023-02361-1 36906613 PMC10008549

[44] Maccora J Peters R Anstey KJ : What does (low) education mean in terms of dementia risk? A systematic review and meta-analysis highlighting inconsistency in measuring and operationalising education. *SSM - Population Health.* 2020;12:100654. 10.1016/j.ssmph.2020.100654 33313373 PMC7721642

[45] Díaz-Venegas C Downer B Langa KM : Racial and ethnic differences in cognitive function among older adults in the USA. *Int. J. Geriatr. Psychiatry.* 2016;31(9):1004–1012. 10.1002/gps.4410 26766788 PMC4945484

[46] Hwangbo S Kim YJ Park YH : Relationships between educational attainment, hypertension, and amyloid negative subcortical vascular dementia: The brain-battering hypothesis. *Front. Neurosci.* 2022;16. 10.3389/fnins.2022.934149 35992915 PMC9388911

[47] Williams BD Pendleton N Chandola T : Does the association between cognition and education differ between older adults with gradual or rapid trajectories of cognitive decline? *Aging Neuropsychol. Cognit.* 2022;29(4):666–686. 10.1080/13825585.2021.1889958 33683174

[48] Gonçalves NG Avila JC Bertola L : Education and cognitive function among older adults in Brazil and Mexico. *Alzheimers Dement.* 2023;15(3):e12470. 10.1002/dad2.12470 37771429 PMC10523452

[49] Alley D Suthers K Crimmins E : Education and cognitive decline in older Americans:Results from the AHEAD Sample. *Res. Aging.* 2007;29(1):73–94. 10.1177/0164027506294245 19830260 PMC2760835

[50] Chu C-S Sun IW Begum A : The association between subjective memory complaint and objective cognitive function in older people with previous major depression. *PLoS One.* 2017;12(3):e0173027. 10.1371/journal.pone.0173027 28267772 PMC5340362

[51] Mitchell AJ : Is it time to separate subjective cognitive complaints from the diagnosis of mild cognitive impairment? *Age Ageing.* 2008;37(5):497–499. 10.1093/ageing/afn147 18682432

[52] Dhana A DeCarli C Dhana K : Association of subjective memory complaints with white matter hyperintensities and cognitive decline among older adults in Chicago, Illinois. *JAMA Netw. Open.* 2022;5(4):e227512–e227512. 10.1001/jamanetworkopen.2022.7512 35426922 PMC9012965

[53] Muhammad T Govindu M Srivastava S : Relationship between chewing tobacco, smoking, consuming alcohol and cognitive impairment among older adults in India: A cross-sectional study. *BMC Geriatr.* 2021;21(1):85. 10.1186/s12877-021-02027-x 33514331 PMC7847155

[54] Medical News Today: Drinking just 3 cans of beer a week may be linked to cognitive decline. 2024. Reference Source

[55] Devere R : The cognitive consequences of alcohol use. 2024. Reference Source

[56] Andriyanto A Rekawati E Rahmadiyah DC : Increasing knowledge, attitudes, skills, and glucose control in type-2 diabetic patients through EMAS interventions. 2019;9(2):10. 10.14710/nmjn.v9i2.22989

[57] Rahmayanti Y : Hubungan lama menderita hipertensi dengan penurunan fungsi kognitif pada lansia. *Jurnal Aceh Medika.* 2018;2(2):241–246. Reference Source

[58] Kim J Park E An M : The cognitive impact of chronic diseases on functional capacity in community-dwelling adults. *J. Nurs. Res.* 2019;27(1):e3–e8. 10.1097/jnr.0000000000000272 29985821 PMC6369881

[59] Pessotti CFC Fonseca LC Tedrus GMAS : Family caregivers of elderly with dementia relationship between religiosity, resilience, quality of life and burden. *Dement. Neuropsychol.* 2018;12:408–414. 10.1590/1980-57642018dn12-040011 30546852 PMC6289474

[60] Wilson RS Segawa E Boyle PA : The natural history of cognitive decline in Alzheimer’s disease. *Psychol. Aging.* 2012;27(4):1008–1017. 10.1037/a0029857 22946521 PMC3534850

[61] Sanjuán M Navarro E Calero MD : Effectiveness of cognitive interventions in older adults: A review. *Eur. J. Investig. Health Psychol. Educ.* 2020;10(3):876–898. 10.3390/ejihpe10030063 34542517 PMC8314287

[62] Hasan F : strobe checklist. *Zenodo.* 2024. 10.5281/zenodo.14048299

