# Supplementary material for: Cognitive function and its determinants in elderly Indonesians residing in long-term care: Insights from a cross-sectional study
Source: F1000Res. 2025 Aug 22;13:1384. Originally published 2024 Nov 18. [Version 2] doi: 10.12688/f1000research.158490.2 (PMC12355162; doi:10.12688/f1000research.158490.2)
Supplement: Peer review history

# Reviewer report: Reviewer response for version 1

**DOI:** 10.5256/f1000research.174085.r395149

**Published:** 2025-08-19

**Authors:** Saranya Pimolkatekul

**Affiliations:** 1 Department of Nursing Administration and Professional Fundamention, Navamindradhiraj University, Bangkok, Thailand

This is a well-written cross-sectional study that investigates the relationship between cognitive function and associated risk factors among elderly Indonesians residing in long-term care settings. The study addresses a relevant public health concern and offers useful descriptive data. However, there are a few minor issues that should be considered to improve the clarity and impact of the paper.

 Firstly, the background would benefit from a clearer articulation of the knowledge gap that this study aims to address. Including a brief summary of what previous studies have not covered particularly in the context of elderly residents in long-term care would better highlight the importance and novelty of this research. Secondly, the manuscript uses various terms interchangeably, such as elderly, older people, and long-term care, long-term care facilities, long-term care institutions. This inconsistency could cause confusion for readers, especially as these terms may have different definitions. To enhance clarity, it is recommended to use consistent terminology throughout the paper. Lastly, while the introduction effectively provides prevalence data and outlines the factors related to cognitive function, it lacks essential details about the study setting and a more explicit justification for focusing on the long-term care population. Clarifying why this particular population and setting were selected would strengthen the rationale for the study.

**Major Comments:**

 Results (Tables): Please clarify and recheck all the numerical values presented in the tables. For example, there are inconsistencies between Table 1 and Table 2 regarding variables such as education level, subjective memory complaints, and living arrangements. In particular, the data on disease history raises concern. Although the total sample size is reported as 350, the frequencies listed appear to account for only 308 participants. It is also unclear how the 148 participants (42.3%) reporting good health are included in this count. Additionally, some p-values appear to be missing in Table 2. Please ensure that all relevant statistical results are reported and that values are consistent across tables.

**Minor Comments:**

 Study Participants: Please provide more context to clarify the inclusion and exclusion criteria, as well as whether a sample size calculation was conducted and how the final sample size was determined.

 Tables: Table 1 appears to present similar information to Table 2, which already provides the detailed results. To avoid redundancy and improve clarity, consider removing Table 1 and retaining only Table 2.

 The conclusion section should more clearly emphasize the main findings of the study, particularly those that are statistically and/or clinically significant. Highlighting the key results will help reinforce the contributions of the study. Additionally, the conclusion should briefly acknowledge the study’s limitations such as the cross-sectional design, potential selection bias, or limited generalizability to provide context for interpreting the results. Including these limitations can also guide future research by suggesting areas that need further exploration or different methodological approaches.

Is the work clearly and accurately presented and does it cite the current literature?

Yes

If applicable, is the statistical analysis and its interpretation appropriate?

I cannot comment. A qualified statistician is required.

Are all the source data underlying the results available to ensure full reproducibility?

Partly

Is the study design appropriate and is the work technically sound?

Yes

Are the conclusions drawn adequately supported by the results?

Yes

Are sufficient details of methods and analysis provided to allow replication by others?

Partly

Reviewer Expertise:

Diabetes Mellitus, Frailty, Older adults

I confirm that I have read this submission and believe that I have an appropriate level of expertise to confirm that it is of an acceptable scientific standard, however I have significant reservations, as outlined above.

# Reviewer report: Reviewer response for version 1

**DOI:** 10.5256/f1000research.174085.r395145

**Published:** 2025-08-14

**Authors:** Rian Adi Pamungkas

**Affiliations:** 1 Universitas Esa Unggul, Jakarta Barat, Indonesia

The topic is highly relevant in light of global population aging and the increasing burden of cognitive decline in older adults. The use of the MoCA-Ina, a culturally adapted and validated instrument, is appropriate and strengthens internal validity. However, several areas require further clarification, elaboration, and strengthening to enhance the manuscript’s scientific rigor and impact

 1. While the study highlights a specific population (elderly in LTCIs), the novelty could be more explicitly framed in the *Introduction* and *Discussion*. How does this study differ significantly from prior regional studies? How does it inform global policy/practice?

 2. The authors used univariate logistic regression followed by multivariate logistic regression. However, a rationale for the model-building strategy is missing. Were interaction effects or multicollinearity assessed?

 3. The explained variance in the logistic regression model (R² = 32.1%) could benefit from more contextual interpretation.

Is the work clearly and accurately presented and does it cite the current literature?

Yes

If applicable, is the statistical analysis and its interpretation appropriate?

Yes

Are all the source data underlying the results available to ensure full reproducibility?

Yes

Is the study design appropriate and is the work technically sound?

Yes

Are the conclusions drawn adequately supported by the results?

Yes

Are sufficient details of methods and analysis provided to allow replication by others?

Yes

Reviewer Expertise:

Gerontology nursing

I confirm that I have read this submission and believe that I have an appropriate level of expertise to confirm that it is of an acceptable scientific standard.

# Reviewer report: Reviewer response for version 1

**DOI:** 10.5256/f1000research.174085.r395151

**Published:** 2025-08-14

**Authors:** Made Satya Nugraha Gautama

**Affiliations:** 1 Department of Nursing, Universitas Pendidikan Ganesha, Bali, Indonesia

The study addresses an important topic: cognitive function in elderly Indonesians in long-term care. This is a growing population with specific needs. The manuscript is generally well-written and organized.

 The findings have potential implications for healthcare practices in long-term care settings.

 However I have several comments/questions should be addressed below:

 - Provide more detail about the "major Long-Term Care Institutions in Jakarta." How many institutions were included?

 - "Comprehensive sampling method" is vague. Was it a census of all eligible residents in the selected facilities? Or a stratified random sample? If not random sampling, explain potential biases.

 - "Older people (≥60 years) devoid of eyesight or hearing impairments" – Why were individuals with eyesight or hearing impairments excluded?

 I believe that addressing these points, particularly in the Methods section, will strengthen the manuscript and enhance its overall impact.

Is the work clearly and accurately presented and does it cite the current literature?

Yes

If applicable, is the statistical analysis and its interpretation appropriate?

Yes

Are all the source data underlying the results available to ensure full reproducibility?

Yes

Is the study design appropriate and is the work technically sound?

Yes

Are the conclusions drawn adequately supported by the results?

Yes

Are sufficient details of methods and analysis provided to allow replication by others?

Yes

Reviewer Expertise:

Adult Nursing, Palliative, Quantitative Research

I confirm that I have read this submission and believe that I have an appropriate level of expertise to confirm that it is of an acceptable scientific standard.

# Reviewer report: Reviewer response for version 1

**DOI:** 10.5256/f1000research.174085.r390862

**Published:** 2025-07-24

**Authors:** Yuni Asri

**Affiliations:** 1 Department of Nursing, Faculty of Health and Science, Institut Teknologi, Sains dan Kesehatan RS dr Soepraoen Kesdam V/Brawijaya, Malang, Indonesia

This robust study used appropriate tools (MoCA-Ina) and valid analyses. To enhance clarity and reproducibility, authors should categorize variables (e.g., age, education), justify their inclusion in regression models, and detail data distribution and missing data handling in tables or text. These improvements would boost transparency and strengthen the study’s reliability.

Is the work clearly and accurately presented and does it cite the current literature?

Yes

If applicable, is the statistical analysis and its interpretation appropriate?

Yes

Are all the source data underlying the results available to ensure full reproducibility?

Yes

Is the study design appropriate and is the work technically sound?

Yes

Are the conclusions drawn adequately supported by the results?

Yes

Are sufficient details of methods and analysis provided to allow replication by others?

Yes

Reviewer Expertise:

public health, prevalence study, epidiomilogy.

I confirm that I have read this submission and believe that I have an appropriate level of expertise to confirm that it is of an acceptable scientific standard.

## References

[1] : Global, regional, and national burden of stroke and its risk factors, 1990–2021: a systematic analysis for the Global Burden of Disease Study 2021. *The Lancet Neurology* .2024;23(10) : 10.1016/S1474-4422(24)00369-7 973-1003 10.1016/S1474-4422(24)00369-7 PMC1225419239304265
